# Making a Case for an Autism-Specific Multimorbidity Index: A Comparative Cohort Study

**DOI:** 10.1007/s10803-025-06823-x

**Published:** 2025-05-16

**Authors:** Dewy Nijhof, Filip Sosenko, Laura McKernan Ward, Deborah Cairns, Laura Hughes, Ewelina Rydzewska

**Affiliations:** 1 School of Health and Wellbeing, University of Glasgow, Glasgow, United Kingdom; 2 Health Informatics Centre, School of Medicine, Ninewells Hospital, University of Dundee, Dundee, DD1 9SY United Kingdom; 3 School of Health in Social Science, University of Edinburgh, Edinburgh, United Kingdom

**Keywords:** Autism Spectrum Disorder, Multimorbidity, Multimorbidity Index, COVID- 19, Mortality, Long-term Conditions

## Abstract

Autistic people experience challenges in healthcare, including disparities in health outcomes and multimorbidity patterns distinct from the general population. This study investigated the efficacy of existing multimorbidity indices in predicting COVID- 19 mortality among autistic adults and proposes a bespoke index, the ASD-MI, tailored to their specific health profile. Using data from the CVD-COVID-UK/COVID-IMPACT Consortium, encompassing England’s entire population, we identified 1,027 autistic adults hospitalized for COVID- 19, among whom 62 died due to the virus. Predictors were selected using logistic regression with fivefold cross-validation, comparing AUCs amongst multimorbidity indices. Diabetes, coronary heart disease, and thyroid disorders were selected as predictors for the ASD-MI, outperforming the Quan Index, a general population-based measure, with an AUC of 0.872 versus 0.828, respectively. Notably, the ASD-MI exhibited better model fit (pseudo-R2 0.25) compared to the Quan Index (pseudo-R2 0.20). These findings underscore the need for tailored indices in predicting mortality risks among autistic individuals. However, caution is warranted in interpreting results, given the limited understanding of morbidity burden in this population. Further research is needed to refine autism-specific indices and elucidate the complex interplay between long-term conditions and mortality risk, informing targeted interventions to address health disparities in autistic adults. This study highlights the importance of developing healthcare tools tailored to the unique needs of neurodivergent populations to improve health outcomes and reduce disparities.

**Supplementary Information:**

The online version contains supplementary material available at https://doi.org/10.1007/s10803-025-06823-x.

## Introduction

Autism Spectrum Disorder (ASD) is a neurodevelopmental disorder characterised mainly by differences in social communication and interaction, and repetitive and restrictive behaviours, including differences in sensory preferences (American Psychiatric Association, 2013). Autistic people are estimated to account for 0.6 to 1.0% of the global population (Salari et al., 2022; Talantseva et al., 2023; Zeidan et al., 2022). They experience substantial health inequalities, including multimorbidity (Bishop-Fitzpatrick & Rubenstein, 2019; Croen et al., 2015), defined as the presence of at least two long-term conditions (LTCs) (NICE | The National Institute for Health and Care Excellence, 2016). ASD has been proposed as a potential risk factor for the development of COVID- 19 infections due to a number of factors including; experiencing difficulties in maintaining social distancing (Brondino et al., 2021), and use of atypical antipsychotics, such as risperidone, frequently prescribed to individuals with ASD (Fusar-Poli et al., 2019), which has been shown to disrupt immune responses (May et al., 2020), and therefore increase the risk of COVID- 19 infection and mortality.

Autistic people have a different pattern of multimorbidity compared to the general population (Hossain et al., 2020a; Rydzewska et al., 2021a) which can result in premature mortality (Forsyth et al., 2023; Tsai et al., 2023). What is more, literature suggests that autistic people are likely to experience poor physical health (Rydzewska et al., 2019, 2021a), high prevalence of multimorbidity (British Medical Association, 2014), and premature mortality (Catalá-López et al., 2022; Forsyth et al., 2023; Hirvikoski et al., 2016). This highlights the necessity of capturing the full burden of multimorbidity to detect poor health and provide suitable care.

Multimorbidity indices, which are used to predict the prognosis of patients based on their medical history or to measure the comorbidity burden, are currently based on the general population (Charlson et al., 1987; Elixhauser et al., 1998; Van Walraven et al., 2009), and may not be applicable to the population with ASD. Multimorbidity indices use previous and current diagnoses as predictors for the outcome of interest, such as quality of life, hospital admissions, healthcare use, and mortality (Diederichs et al., 2011; Stirland et al., 2020). Alternatively, the index is used to control for long-term conditions or multimorbidity while studying other associations.

A variety of multimorbidity indices exist, and characteristics amongst these instruments vary in terms of included LTCs, weighting of LTCs and even outcome measures (Diederichs et al., 2011). The most used and well-known index is the Charlson Comorbidity Index (CCI) which uses scores for 17 LTCs to predict the ten-year risk of death within one year after hospital admission (Charlson et al., 1987). Since its conception, several modifications of the CCI have been created, ranging in which LTCs are included, total number of diseases included, weights of individual LTCs and overall score construction (Diederichs et al., 2011). The most well-known modifications include the Elixhauser Index (including 30—or, for some variants, 31—LTCs) (Elixhauser et al., 1998) and the van Walraven (VW) variant of the Elixhauser Index, which includes a weighted summary score for streamlined use, based on the 30 LTCs from the Elixhauser Index (Van Walraven et al., 2009). A more recent and streamlined mortality prediction tool is the Quan Index, which incorporates 12 LTCs derived from International Classification of Diseases, 10 th Revision (ICD- 10) codes (Quan et al., 2011). A notable feature of the Quan Index is its construction using up-to-date population health data from six countries, which informed the weighting of LTCs to improve predictive accuracy in healthcare settings (Quan et al., 2011). While the Charlson Index is a foundational tool for mortality prediction and has been widely adapted, its weights and included conditions were designed for general hospitalised populations. The Quan Index, in contrast, was chosen for this study because its streamlined approach focuses specifically on in-hospital mortality and utilises more recent datasets. Further support for the use of the Quan Index rather than the Charlson Index comes from the fact that some conditions included in the Charlson Index are not as frequently observed in autistic individuals, such as HIV/AIDS and metastatic solid tumours (Cassell et al., 2018; Croen et al., 2015).

Most indices use scores to estimate prognosis, with higher scores usually being indicative of more severe risk of death. These scores are usually additive, where the presence of multiple LTCs will lead to a higher score. Scores are based on weights for each condition, which usually are derived from the modelling of the risk, in which the weight quantifies their contribution towards the outcome. The long history of development and validation of these instruments has resulted in establishing strong evidence of associations between multimorbidity and mortality risk, decline in physical and mental functioning, and quality of life (Diederichs et al., 2011; Stirland et al., 2020). However, less research has focused on the selection criteria for inclusion of LTCs and most indices are constructed for the general population (Diederichs et al., 2011).

As common physical and mental LTCs differ in the population with ASD compared from those in the general population (Forsyth et al., 2023; Hossain et al., 2020b; Rydzewska et al., 2021b), this may be of importance. For example, autistic people have a high burden of mental health conditions such as anxiety, depression and schizophrenia (Hwang et al., 2019; Tsai et al., 2023). Autistic people are also more likely to experience co-occurring physical conditions, such as epilepsy, autoimmune disorders and obesity (Hwang et al., 2019; Tsai et al., 2023). Therefore, the effectiveness and sensitivity of the established multimorbidity indices for the prediction of health outcomes in the autistic population and subsequent treatment based on those predictions warrants an investigation into their validity. Indeed, studies examining the association between LTCs and mortality in autistic people make use of established multimorbidity indices, such as the CCI, but include LTCs known to be prevalent in the autistic population (Hwang et al., 2019; Tsai et al., 2023). These studies found that the included LTCs, such as epilepsy, mental health conditions (e.g., bipolar disorder, schizophrenia, major depressive disorder) or intellectual disabilities carried higher risk of death in the autistic population (Hwang et al., 2019; Tsai et al., 2023). This suggests that traditional indices may not accurately reflect the health profile or capture the full extent of multimorbidity and its concurrent risk for the autistic population.

To our knowledge, there has been no previous research into the effectiveness of using existing multimorbidity indices to investigate health outcomes in autistic people nor does a multimorbidity index specifically tailored for autistic populations exist. The primary objective of this study was to investigate the efficacy and sensitivity of an established multimorbidity index for the general population, the Quan Index, compared to a bespoke index, hereafter referred to as the Autism Spectrum Disorder Multimorbidity Index (ASD-MI), in predicting mortality from COVID- 19 within the autistic population. This study represents a supplementary analysis derived from a broader investigation examining COVID- 19 outcomes in autistic adults (Nijhof et al., n.d.-b; Sosenko et al., 2023). As such, the study design employed a convenience sampling approach, necessitated by the availability of relevant data sources.

## Methods

### Data Sources

This was part of a larger cross-sectional study using a whole-country population covering England to investigate mortality rates due to COVID- 19 (Nijhof et al., n.d.-a; Sosenko et al., 2023). It was conducted on behalf of, and accessed data made available in NHS England’s Secure Data Environment service for England, the CVD-COVID-UK/COVID-IMPACT Consortium (coordinated by the BHF Data Science Centre). The datasets used for the current study were Hospital Episode Statistics (HES), COVID- 19 Hospitalisation in England Surveillance System (CHESS), Second Generation Surveillance System (SGSS), General Practice Extraction Service (GPES) Data for Pandemic Planning and Research (GDPPR) and Civil Registry Deaths from the Office for National Statistics (ONS-D). All but the last of these datasets are updated through the National Health Service (NHS) and processed by NHS England who then opens up the data for secondary use. The ONS-D dataset is updated through the ONS. Data from 1 March 2020 up to and including 31 December 2021 was used. Data linkage was conducted using an anonymised version of the NHS number (a ten-digit number used as a unique identifier within the UK NHS to identify patients). Ethical approval was obtained from the CVD-COVID-UK/COVID-IMPACT Consortium and the University of Glasgow Ethics Committee.

Autistic people were identified using SNOMED concepts sourced from the GDPPR and HES ‘Autism Diagnosis Codes’ cluster^1^ (cluster AUTISM COD version 2021/12/21). With regards to the long-term conditions considered as ‘candidates’ for the ASD-MI, thirty-five long-term conditions common to the UK population were identified (Cassell et al., 2018) (see Supplementary materials, Table 1). One-year look-back window (in HES) was used to identify patients with a given condition, counting from the date of COVID- 19 admission.

Demographic (age, sex, ethnicity, Index of Multiple Deprivation (IMD)) and LTCs were explored using percentages of total autistic population. IMD decile is a measure used in the United Kingdom to assess relative deprivation across different areas or regions. The IMD ranks from 1 (most deprived) to 10 (least deprived) based on several indicators of deprivation, such as income, employment, education, health, crime, and living environment. In our analyses, instead of having 10 distinct deciles, the IMD rankings were grouped into 5 categories, each representing a range of deprivation levels. This grouping simplifies the interpretation of deprivation levels and facilitates comparisons between areas.

The decision to compare our ASD-MI to the Quan Index rather than the Charlson Index was carefully considered. Firstly, our study was constrained by the requirement to use ICD- 10 code mapping, which is a feature supported by the Quan Index but not the Charlson Index (Quan et al., 2011). Furthermore, the Quan Index’s more recent update and incorporation of contemporary population health data from six countries provided a distinct advantage (Quan et al., 2011). This up-to-date information enhances the predictive capabilities of the Quan Index, aligning with our study’s objective of accurately predicting in-hospital mortality risk among autistic adults with COVID- 19. To predict in-hospital mortality, the Quan Index includes 12 LTCs instead of the original 17 used in the Charlson Index to construct weights (Quan et al., 2011) (see Supplementary materials, Table 2). Again, a one-year look-back window in HES was used to calculate the patient’s Quan Index score.

### Population

The study sample was drawn from a larger study sample that covered the whole population of England who were alive on 1 January 2020, which focused on rates of COVID- 19 infection, hospitalisation and mortality in autistic adults (Nijhof et al., n.d.-a). Adults who had a primary care diagnosis of autism and who were hospitalised for COVID- 19 (defined as any CHESS record, or a HES record with an ICD- 10 code of U07.1 or U07.2 recorded in any position on the admission diagnosis). ‘COVID- 19 cause of death’ was defined as a death that occurred within 28 days from the first day of admission and where COVID- 19 was mentioned on the death certificate (either primary or other contributing causes using ICD- 10 code U07.1 or U07.2). For the larger study, the total sample with confirmed COVID- 19 was 32,372 autistic adults (see Table 1). The sample used to construct the multimorbidity index consisted of 1,027 autistic adults hospitalised for COVID- 19, of which 62 died due to COVID- 19. Data up to 31 December 2021 was included. Notably, this timeframe encompassed both the pre-vaccination phase of the pandemic as well as the post-vaccination period. Separate analysis of pre- and post-vaccination periods was not possible due to small sample sizes.

**Table 1:** Demographic and health characteristics of autistic adults hospitalised for COVID- 19

	Covid- 19 hospitalised adults who subsequently died from covid	Covid- 19 hospitalised adults who subsequently did not die from covid	All covid- 19 hospitalised adults
Total N	62	965	1,027
Demographic information			
Age (median, IQR)	63.4 [48.9, 75.2]	31.1 [23.6, 50.0]	32.3 [24.0, 53.0]
Female (%)	19.4	40.6	39.3
IMD decile (mean)	4.8	4.6	4.6
Ethnicity (%)			
White	96.8	89.5	90.0
Asian	< 10 CASES	3.2	3.1
Black	< 10 CASES	2.7	2.5
Mixed	< 10 CASES	2.7	2.6
Other	< 10 CASES	1.5	1.4
Long-term conditions (%)			
Count of LTC (mean)	11.0	7.4	7.6
Alcohol problems	33.9	31.3	31.5
Anorexia or bulimia	< 10 CASES	8.3	8.1
Anxiety & other neurotic	64.5	58.8	59.1
Asthma (currently treated)	27.4	27.9	27.9
Atrial fibrillation	33.9	10.4	11.8
Blindness and low vision	< 10 CASES	6.7	7.0
Bronchiectasis	< 10 CASES	< 10 CASES	1.0
Cancer—[New]Diagnosis in last five years	40.3	18.0	19.4
Coronary heart disease	58.1	19.6	21.9
Chronic kidney disease	< 10 CASES	1.2	1.2
Chronic Liver Disease and Viral Hepatitis	43.6	32.5	33.2
COPD	24.2	10.0	10.8
Dementia	51.6	52.5	52.5
Depression	38.7	43.0	42.8
Diabetes	85.5	37.8	40.7
Diverticular disease of intestine	43.6	25.6	26.7
Epilepsy (currently treated)	43.6	21.6	22.9
Heart failure	61.3	22.7	25.0
Hearing loss	< 10 CASES	6.1	6.6
Hypertension	66.1	24.6	27.1
Inflammatory bowel disease	17.7	14.6	14.8
Irritable bowel syndrome	< 10 CASES	9.3	9.4
Migraine	< 10 CASES	5.5	5.7
Multiple sclerosis	< 10 CASES	< 10 CASES	< 10 CASES
Peptic Ulcer Disease	< 10 CASES	10.8	10.9
Parkinson’s disease	< 10 CASES	2.1	2.3
Prostate disorders	< 10 CASES	6.8	7.2
Psychoactive substance misuse (not alcohol)	64.5	62.3	62.4
Psoriasis or eczema	< 10 CASES	7.2	7.2
Peripheral vascular disease	22.6	7.2	8.1
Rheumatoid arthritis	22.6	15.2	15.7
Schizophrenia (and related non-organic psychosis) or bipolar disorder	83.9	78.9	79.2
Chronic sinusitis	< 10 CASES	1.4	1.4
Stroke & transient ischaemic attack	62.9	51.1	51.8
Thyroid disorders	17.7	5.6	6.3

### Data Analysis

All data analysis was conducted in Python 3.7 and SQL. The analysis was performed according to a pre-specified analysis plan published on GitHub, along with the phenotyping and analysis code (https://github.com/BHFDSC/CCU030_03).

#### Feature Selection

The ASD-MI was constructed by applying a forward variable selection procedure to identify which of the conditions should be included in the index. This was done through logistic regression with k-fold cross-validation (k = 5). K-fold cross-validation splits the sample into k subsamples, where each subsample can be used for either training or testing the model. The model was estimated based on the pooled data from k- 1 samples, then validated in the fifth holdout sample for validation. This process was repeated until every sample had served as the validation sample.

The initial feature set included all 35 long-term conditions (see Supplementary materials, Table 3) with age included as an addition predictor to align with the methodology used to create the CCI (Charlson et al., 1987). To select the predictors, a logistic regression model was iteratively fitted to predict COVID- 19 death (indicated by presence or absence). At each iteration, one predictor was added to the model to test for inclusion, as measured by improvements in Area Under the Curve (AUC). The selection process was halted when the AUC improvement was less than 0.001 upon adding a new variable.

The final model with the most predictive long-term conditions then formed the basis of the ASD-MI, with the coefficients of the selected long-term conditions from that model serving as the individual weight for each of the conditions, with the final ASD-MI score as a weighted sum of the selected conditions.

#### Comparison Against Quan Index

Finally, predictive performance of the ASD-MI was then compared to that of the Quan index. Again, predictive performance was assessed for both the Quan Index and the ASD-MI through fivefold cross-validation with AUC as evaluation metric and logistic regression models with the absence or presence of COVID- 19 death as the outcome. Age was again included as control variable.

## Results

### Demographics

The results presented in Table 1 provide a comprehensive overview of the demographic and health characteristics of autistic adults hospitalised for COVID- 19, categorised based on subsequent COVID- 19 mortality outcomes. Among the total cohort of 1,030 hospitalised adults, 60 individuals died due to COVID- 19, while 965 individuals did not. The median age of those who died from COVID- 19 was notably higher at 63.4 years (interquartile range: 48.9–75.2) compared to survivors, with a median age of 31.1 years (interquartile range: 23.6–50.0). A higher proportion of females were observed among survivors (40.6%) compared to those who died from COVID- 19 (19.4%). Additionally, the mean IMD decile was similar between both groups, indicating comparable levels of deprivation. Regarding LTCs, individuals who subsequently died from COVID- 19 had a higher mean count of LTCs (11.0) compared to survivors (7.4). Furthermore, several LTCs demonstrated notable differences in prevalence between the two groups, with higher proportions observed among individuals who died from COVID- 19, although these results should be interpreted with caution due to small numbers.

### Predictor Selection for the ASD-MI

By employing a forward variable selection procedure using logistic regression with fivefold cross-validation, we systematically evaluated the predictive capabilities of 35 long-term conditions (LTCs) in relation to COVID- 19 mortality risk among autistic adults. This approach allowed us to identify the most influential LTCs associated with the outcome of interest. By iteratively assessing the model’s performance using the Area Under the Curve (AUC) score as the evaluation metric, we identified three LTCs that consistently demonstrated the highest predictive power. The selected variables for the ASD-MI were diabetes, coronary heart disease and thyroid disorders. These LTCs emerged as significant contributors to the ASD-MI, reflecting their strong association with COVID- 19 mortality risk in this population. This process ensured that the selected LTCs were robust indicators of mortality risk, disregarding indicators that did not add to the predictive power of the model. Table 2 shows the coefficients of the LTCs included in the ASD-MI and their corresponding Odds Ratios. Using the coefficients from the model, the formula for the Multimorbidity Index (MI) was:

$${\mathrm{MI}}\, = \,1.21*{\mathrm{DIA}}\, + \,0.71*{\mathrm{CHD}}\, + \,0.67*{\mathrm{THY}}$$

Where DIA refers to diabetes, CHD to coronary heart disease, and THY to thyroid disorders. Each are multiplied by their respective coefficients from the model.

**Table 2:** Logistic Regression results for the models using the Quan Index and the ASD-MI

	Odds Ratio	95% Confidence Interval	P-value
Model with Quan Index			
Age at COVID	1.06	1.05, 1.08	0.000
Quan Index score	1.16	0.98, 1.37	0.081
Constant	0.00	0.00, 0.01	0.000
Model with ASD-MI			
Age (at COVID)	1.05	1.03, 1.07	0.000
Multimorbidity Index score	2.72	1.78, 4.14	0.000
Constant	0.00	0.00, 0.01	0.000

### Comparative Predictive Performance of the Two Models

The logistic model using the ASD-MI had a higher AUC value of 0.872 (95% CI: 0.867–0.877) than the model using the Quan Index, which had an AUC value of 0.828 (95% CI: 0.821–0.835). Table 2 shows the regression results for both models using the Quan Index and the ASD-MI. Further, model fit is better for the model using the ASD-MI (pseudo-R^2^ 0.25) than for the model using Quan Index (pseudo-R^2^ 0.20).

## Discussion

This feasibility study aimed to investigate whether there is a need for a specially tailored multimorbidity index to predict COVID- 19-related mortality in autistic adults. Using LTCs that more accurately describe the health profile of the autistic population, the results show that our ASD-MI is moderately better at predicting the risk of COVID- 19 death amongst autistic adults than a general population-based index. This study is a case in point to show the potential for development for general poor health outcomes specific to autistic adults, especially if trained for mortality outside of a COVID- 19 context.

In the broader context of COVID- 19 mortality and multimorbidity, previous research from our larger study (using the full sample as discussed in the methods) has identified key predictors of severe outcomes in autistic individuals. This included age, vaccination status, and the presence of complex multimorbidity, with the latter being significantly more prevalent among autistic adults. Notably, while the impact of age remains a critical factor in mortality risk, it was less pronounced in the autistic population compared to the general population despite increased Age Standardised Mortality Rates (ASMRs) (2020: ASMR = 278.9 (95% confidence interval (CI) [278, 278] for autistic adults, ASMR = 166.2, 95% CI [166, 166] for general population; 2021: ASMR = 271.5, 95% CI [271, 271] for autistic adults, ASMR = 143.1, 95% CI [143, 143] for general population) [blinded reference]. The study found that interaction between multimorbidity and polypharmacy may play a role in influencing COVID- 19 outcomes, though effect sizes for major risk factors were broadly similar between autistic and non-autistic individuals despite increased ASMRs for the autistic population, suggesting future research on complex multimorbidity and polypharmacy is warranted in the autistic population. Further details on overall mortality, hospitalization, and multimorbidity patterns can be found in [blinded reference].

While these broader findings are informative, the current study focused on the potential of the ASD-MI to predict COVID- 19 related mortality in autistic adults. Our results suggest a better prediction of COVID- 19 mortality in autistic adults using the three predictors of diabetes, coronary heart disease and thyroid dysfunction, though caution should be used when interpreting these results. We emphasise that further research is needed to accurately predict all-cause mortality due to the known associations of these three conditions with COVID- 19 (Saha et al., 2021; Zhang et al., 2021; Zhou et al., 2021). Current evidence on all-cause mortality in autistic individuals points mainly to external causes, such as suicide, and neurological disorders (Forsyth et al., 2023; Tsai et al., 2023) It could well be that the current study’s formulation of the ASD-MI does not generalise well to all-cause mortality in autistic adults. Furthermore, relatively little is known about the morbidity burden of coronary heart disease and thyroid disorders in autistic individuals. This further emphasises the importance of studying the general health profile of autistic individuals and how a specifically tailored multimorbidity index could help in finding target LTCs for future research. Nevertheless, while the ASD-MI was developed and tested specifically within the context of COVID- 19, its potential application extends to similar scenarios involving novel infectious diseases or other public health crises. The predictors identified—diabetes, coronary heart disease, and thyroid disorders—may serve as an initial framework for future risk assessments. However, additional research is needed to confirm their broader applicability and relevance outside the specific context of the COVID- 19 pandemic. Continued refinement and validation are necessary to enhance preparedness and improve outcomes for autistic individuals in future risk situations.

One aspect of the study that needs to be emphasised is that our ASD-MI, similar to the original CCI and Quan Index, was developed purely for the purpose of prediction. Accordingly, predictive power was the only criterion for selecting one model over another. Therefore, like the creators of earlier indices we did not aim to explain why selected LTCs were better predictors than LTCs that were ruled out. And we do not seek to justify LTC selection on grounds of existing knowledge and risk factors for COVID- 19 mortality (see (Shmueli, 2010) for further discussion).

The current study did find significant differences in the predictive value of our ASD-MI and a standard index, despite limiting the data to COVID- 19-related deaths in the peak of the global pandemic in England. Future research expanding this methodology to all-cause mortality using a larger window of time may yield larger sample sizes and more generalisable results. This would arguably help improve predicting mortality for autistic adults in healthcare settings. Further, it would help elucidate the multimorbidity profile of autistic adults by virtue of its selection criteria for included LTCs. Furthermore, these findings and future exploration of all-cause mortality may highlight LTCs and combinations of LTCs that are of most urgency to investigate in order to address increased mortality in autistic adults.

## Strengths and Limitations

This study has several strengths. It is the first study to investigate and construct a multimorbidity index specifically aimed at autistic adults. Foremost, the utilization of a whole-country population encompassing both autistic individuals and the general population ensures a comprehensive and representative sample, effectively mitigating potential sampling biases. Additionally, the study leverages validated datasets, further fortifying the credibility of the results. Furthermore, the inclusion criteria for autism diagnosis are meticulously defined based on clinical diagnoses, incorporating a wide range of older and newer diagnostic codes pertinent to autism. This comprehensive approach enables the study to capture a diverse spectrum of individuals on the autism spectrum, thereby offering a more nuanced and accurate representation of the autistic population in England. Overall, these strengths collectively underscore the study’s capacity to provide valuable insights into the health profiles and multimorbidity patterns among autistic individuals, contributing to a deeper understanding of their healthcare needs.

One potential limitation stems from the sample characteristics. Given the focus on evaluating the multimorbidity index within the context of COVID- 19, the sample composition may be skewed, particularly in terms of age and sex distribution. Notably, while older age has been associated with increased COVID- 19 mortality risk, the autistic sample in this study tended to be younger due to the higher rates of autism diagnosis in children and young individuals. Moreover, the male predominance in both COVID- 19 cases and autism diagnoses introduces further complexity, potentially influencing the observed outcomes. These inherent biases may limit the extrapolation of study findings to broader populations. However, despite these limitations, this study contributes valuable insights into the health profiles of autistic individuals and underscores the importance of developing tailored tools to address their unique healthcare needs, thereby highlighting avenues for further research and exploration in this area.

Another potential limitation is the possible introduction of an inherent bias in the data due to the use of several code mappings to accommodate information coding in the different types of datasets and analysis tools used which originates from data linkage using multiple systems that use different codes within their electronic health records.

Further, while the follow-up time in the CCI and Quan Index is one year after admission, the current study used a follow-up time of 28 days in order to be consistent with the official definition of COVID- 19 death.

## Conclusion

This point in case study demonstrated that the use of our ASD-MI for the adult autistic population provides more accurate predictions of COVID- 19 mortality in this population than a multimorbidity index based on the general population. It shows that application of the ASD-MI trained on all-cause mortality the autistic population may be more appropriate than the use of multimorbidity indices based on the general population and could be used to elucidate key differences between the health profiles of autistic and non-autistic people.

## Supplementary Information

Below is the link to the electronic supplementary material.

Supplementary file1 (DOCX 17 kb)

## Data Availability

The data used in this study are available in NHS England’s Secure Data Environment (SDE) service for England, but as restrictions apply they are not publicly available (50). The CVD-COVID-UK/COVID-IMPACT programme led by the BHF Data Science Centre (51) received approval to access data in NHS England’s SDE service for England from the Independent Group Advising on the Release of Data (IGARD) (52) via an application made in the Data Access Request Service (DARS) Online system (ref. DARS-NIC- 381078-Y9 C5 K)(53). The CVD-COVID-UK/COVID-IMPACT Approvals & Oversight Board (51) subsequently granted approval to this project to access the data within NHS England’s SDE service for England. The de-identified data used in this study were made available to accredited researchers only. Those wishing to gain access to the data should contact bhfdsc@hdruk.ac.uk in the first instance. For the purpose of open access, the authors have applied a Creative Commons Attribution (CC BY) licence to any Author Accepted Manuscript version arising from this submission.

## Footnotes

1 Primary Care Domain Reference Set Portal. NHS Digital, https://digital.nhs.uk/data-and-information/data-collections-and-data-sets/data-collections/quality-and-outcomes-framework-qof/quality-and-outcome-framework-qof-business-rules/primary-care-domain-reference-set-portal (accessed 8 August 2023).
