# Supplementary material for: Making a Case for an Autism-Specific Multimorbidity Index: A Comparative Cohort Study
Source: J Autism Dev Disord. 2025 May 16;56(11):4429–37. doi: 10.1007/s10803-025-06823-x (PMC13687244; doi:10.1007/s10803-025-06823-x)
Supplement: Supplementary file 1 [file 10803_2025_6823_MOESM1_ESM.docx]

# **Supplementary Materials**

*Table 1. List of 35 long-term conditions used as candidates for the ASD-MI.*

Alcohol problems

Anorexia or bulimia

Anxiety & other neurotic

Asthma (currently treated)

Atrial fibrillation

Blindness and low vision

Bronchiectasis

Cancer - [New]Diagnosis in last five years

Coronary heart disease

Chronic kidney disease

Chronic Liver Disease and Viral Hepatitis

COPD

Dementia

Depression

Diabetes

Diverticular disease of intestine

Epilepsy (currently treated)

Heart failure

Hearing loss

Hypertension

Inflammatory bowel disease

Irritable bowel syndrome

Migraine

Multiple sclerosis

Peptic Ulcer Disease

Parkinson’s disease

Prostate disorders

Psychoactive substance misuse (not alcohol)

Psoriasis or eczema

Peripheral vascular disease

Rheumatoid arthritis

Schizophrenia (and related non-organic psychosis) or bipolar disorder

Chronic sinusitis

Stroke & transient ischaemic attack

Thyroid disorders

*Table 2. List of the 12 long-term conditions included in the Quan Index.*

Myocardial Infarction

Congestive Heart Failure

Peripheral Vascular Disease

Cerebrovascular Disease

Hemiplegia or paraplegia

Dementia

Chronic Pulmonary Disease

Rheumatologic Disease

Peptic Ulcer Disease

Diabetes without Chronic Complications

Diabetes with Chronic Complications

Renal Disease

Any Malignancy, including Leukemia and Lymphoma

Metastatic Solid Tumor

Mild Liver Disease

Moderate or Severe Liver Disease

AIDS/HIV

Table 3. Different combinations of the selected LTCs included in the Multimorbidity Index with their respective coefficients and odds ratio.

| **Predictors** | **Multimorbidity Index** | **Odds ratio** |
| --- | --- | --- |
| None of DIA, CHD, THY | 0 | (base) |
| DIA | 1.21 | 3.35 |
| CHD | 0.71 | 2.03 |
| THY | 0.67 | 1.96 |
| DIA, CHD | 1.92 | 6.82 |
| DIA, THY | 1.88 | 6.55 |
| CHD, THY | 1.38 | 3.97 |
| DIA, CHD, THY | 2.59 | 13.33 |
